# Impact of Imaging Protocols on Convolutional Neural Network-Based Pressure Injury Detection

**DOI:** 10.21203/rs.3.rs-7263214/v1

**Published:** 2025-10-21

**Authors:** Miriam Asare-Baiden, Sharon Eve Sonenblum, Kathleen Jordan, Glory Tomi John, Andrew Chung, Judy Wawira Gichoya, Vicki Stover Hertzberg, Joyce C. Ho

**Affiliations:** 1 Emory University, Department of Computer Science and Informatics, Emory University,Atlanta, 30322, USA; 2 Emory University, Nell Hodgson Woodruff School of Nursing, Atlanta, 30322, USA; 3 Emory University, Department of Radiology and Imaging Sciences, School of Medicine,Atlanta, 30322, USA

## Abstract

Pressure injuries remain a critical concern in clinical care, with early detection essential for preventing progression and reducing morbidity. While thermal imaging has demonstrated promise for early pressure injury detection, the impact of imaging protocol variations and patient skin tone on detection accuracy remains underexplored. In this study, we systematically assess how variations in lighting, camera distance, patient positioning, and camera type influence deep learning model performance for early pressure injury detection using both optical and thermal images. A total of 1680 images were collected from 35 healthy adults across diverse skin tones using a factorial design of 12 imaging protocols in a controlled environment where localized cooling was induced to simulate temperature changes. Three deep learning model architectures (MobileNetV2, InceptionNetV3, ResNet50) were evaluated to assess protocol robustness. Thermal imaging significantly outperformed optical imaging, achieving >90% accuracy across models with minimal sensitivity to protocol variations. In contrast, optical performance varied substantially across protocols (32–55% accuracy), demonstrating significant protocol dependency that could impact clinical implementation. Cross-subject error analysis revealed that models focused on both the cool and warm regions in the images, suggesting that current static labeling approaches may be inadequate for dynamic thermal imaging applications. These findings establish the robustness of deep learning models trained on thermal imaging data across diverse skin tones and imaging conditions, providing a critical foundation for future clinical validation in pressure injury detection applications.

## Introduction

Pressure injuries, also known as pressure ulcers, bedsores, or decubitus ulcers, represent a significant healthcare challenge with profound implications for patient outcomes and healthcare systems worldwide. These localized injuries to the skin and underlying tissue result from prolonged pressure, shear, friction, or a combination of these mechanical factors, which usually develop over bony prominences or in areas where medical devices contact the skin.^1^ The economic burden of pressure injuries is substantial and multifaceted. In the United States alone, hospital-acquired pressure injury costs exceed $26.8 billion annually.^2^ Individual treatment costs vary dramatically based on severity, with a single hospital-acquired pressure injury episode potentially costing hospitals anywhere from $500 to more than $70,000.^3^ Beyond direct treatment costs, pressure injuries contribute to extended hospital stays, increased nursing care requirements, legal liability, and reduced quality of life.^4,5^ Estimates suggest that approximately 60,000 deaths occur annually in the United States from sepsis related to pressure injury.^3^

Early detection of pressure injuries is critical for preventing progression to more severe stages and reducing associated morbidity and costs. The earliest signs of pressure injury development include non-blanchable erythema of intact skin, localized warmth or coolness, changes in tissue consistency (firmness or sponginess), and patient reports of pain or unusual sensations in at-risk areas.^1,6^ However, visual inspection, which is a widely used assessment method has significant limitations, particularly in patients with darker skin tones, where temperature changes, subsequent erythema and deep tissue changes preceding visible surface manifestations^7^ may be difficult to detect. Subepidermal moisture measurement^8,9^ and ultrasound^10^ are alternate approaches to detecting pressure injuries but costs of such devices may prevent system-wide implementation.^11,12^

Thermal imaging, using long-wave infrared thermography, has shown promise in skin assessment of deep tissue pressure injuries,^13,14^ aligning with clinical definitions that pain and temperature changes often precede skin color changes. Studies have shown that thermal imaging can identify temperature differences associated with deep tissue pressure injury, with early detection protocols demonstrating significant reductions in hospital-acquired pressure injury rates of up to 60%^13^ and enabling earlier intervention before visual identification is possible.^15^

Previous research has definitively shown that imaging conditions significantly affect the accuracy of pressure injury documentation. Rennert *et al*.^16^ showed that the same wound when captured with proper photographic techniques (image taken with flash light and planes of the wound parallel to the camera lens) measured 20.1*cm*^2^, while with improper techniques, it measured 13.1*cm*^2^ resulting in a 35% underestimation of the true area due to a simple change in the angle of capture. Large variations between images result from multiple factors: changes in camera-to-wound distance (causing scale variance), altered ambient lighting conditions, poor image quality, and differences in camera angle.^17^

Studies have also established that to ensure the visual quality of photographs, research staff must receive intensive training with emphasis on proper focus and lighting^18^, and that standardized protocols require the same light source, intensity, and angle, with the camera at the same angle, distance, rotation and height from the wound.^19,20^ Despite this evidence, the impact of specific imaging variables on pressure injury detection remains incompletely characterized.^21^

While recent studies developed deep learning models for pressure injury detection using thermal imaging,^22,23^ achieving mean average precision values ranging from 76.9% to 90.8%,^24^ none have systematically evaluated how variations in imaging protocols affect model performance for pressure injury detection. Instead, they focus on algorithm development under controlled conditions, without examining the robustness to real-world imaging variations that are common in clinical settings. In addition, none of these studies have evaluated the impact of these protocols on various skin tones and whether specific protocols should be followed when capturing optical and thermal images for early detection of pressure injury.

This study aims to systematically evaluate the impact of imaging protocol variations on the reliability of deep learning models trained to detect temperature change. We assess how lighting conditions, patient positioning, camera distance, and camera type affect deep learning model performance across diverse skin tones using optical and thermal imaging modalities, with controlled cooling applied to simulate the temperature changes that precede early pressure-related tissue damage.

## Methods

### Data Collection

Our study focused on inducing external temperature changes on the patient to assess the impact of imaging protocol, especially in individuals with darker skin tones. This controlled setup isolates temperature changes and assesses whether thermal and optical imaging can reliably detect such changes, with localized cooling serving as a proxy for the early temperature changes associated with pressure injury development. This study recruited participants from March to June, 2024 to collect optical and thermal images from 35 health adults in a controlled simulation environment. Ethical approval for the study was obtained from the Emory University Institutional Review Board (eIRB number 00005999). Written informed consent was obtained from the participants prior to the data collection, and all experiments were performed in accordance with all guidelines and regulations of the Emory University Institutional Review Board. Participants were characterized using the Monk Skin Tone (MST) scale,^25^ a 10-point classification system, by measuring the skin tone at the inner forearm. Recruitment initially targeted 30 participants with MST scores of 6 or higher to ensure adequate representation across darker skin tones, with an additional 5 participants with MST scores of 1–5 subsequently recruited to provide balance. For analytical purposes, the original MST ≥6 group was split into two subgroups: MST 6 Group (n=7, 20%) and MST 7–10 Group (n=23, 65.7%), while the control participants formed the MST 1–5 Group (n=5, 14.3%). The distribution across MST levels and groups is shown in Table 1.

**Figure 1.**
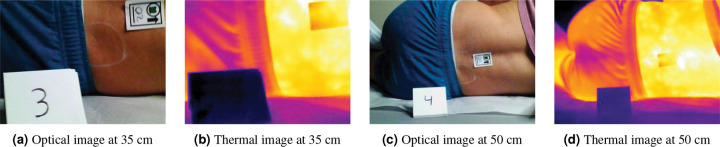
Control images taken with the FLIR E8XT camera in the same position of knee forward and ring lighting, but at different distances, 35 cm versus 50 cm, or Protocol 3 and 4, respectively.

Prior to image collection, we placed a sticker on the lower back to facilitate optical and thermal image alignment and marked two parallel 2” circles (left and right side) on the posterior superior iliac spine of each participant. Temperature changes were induced at the right circle by placing a stone cylinder cooled to 60°C via water bath on this region for 5 minutes. We captured images using two cameras: the FLIR E8XT (640 × 480 optical resolution, 320 × 240 thermal resolution) and the FLIR ONE^®^ Pro (1440 × 1080 optical resolution, 160 × 120 thermal resolution). The image capture protocol followed a factorial design with two lighting conditions (ambient and ring light), two distances (35 and 50 cm), and three postures (forward placement of the top knee, stacked knees, backward placement of the top knee), resulting in 12 different imaging configurations as shown in Table 2. The order of these 12 configurations was randomly assigned and varied between participants. For each configuration and camera, we captured paired images before and after the cooling protocol, yielding 48 images per participant and 1,680 images total (840 control, 840 cooling). Figure 1 shows a series of control images for both modalities captured at different distances with consistent camera positioning and lighting conditions.

### Convolutional Neural Networks

Convolutional neural networks (CNNs) have become the standard approach for medical image analysis^26^. Popular CNN architectures including MobileNets^27^, InceptionNets^28^ and ResNets^29^ are widely used for image analysis. These networks, initially trained on ImageNet’s 1.4 million images across 1,000 object categories, have achieved state-of-the-art performance in medical imaging through transfer learning. This approach leverages knowledge from general object recognition (encoded by utilizing the pre-trained model weights) and applies it to specialized medical domains by fine-tuning the CNN to the domain-specific task. This is particularly valuable as fine-tuning requires less samples given the limited availability of large, annotated clinical datasets. CNNs have emerged as promising tools for pressure injury detection, with studies demonstrating the effectiveness of diverse architectures including VGG-16 and ResNet-50 for wound classification,^30,31^ encoder-decoder networks such as U-Net for precise lesion segmentation^32^ and modern efficient architectures like MobileNet for pressure injury segmentation.^22^ We assessed the sensitivity of three popular CNN architectures, MobileNetV2, ResNet50, and InceptionNetV3, to the image protocol category on detecting whether the image was captured during the control or cooling period.

#### Model Implementation.

We preprocessed all thermal images to ensure the same color corresponded to the same temperature across subjects. We then followed the standard image preprocessing technique used for the original ImageNet specification for each pre-trained architecture. MobileNetV2 and ResNet50 were trained on images of 224 × 224, while InceptionNetV3 used images of size 299 × 299 pixels. All image resizing was performed using bilinear interpolation within the PyTorch framework. To enhance model generalization and mitigate overfitting given the limited number of images, data augmentation was applied during fine-tuning. Specifically, we introduced horizontal flips, vertical flips, and rotation at 20° for each training image.

Fine-tuning for each CNN architecture was performed using the Adam Optimizer with a learning rate of 0.001, batch size of 32, and binary cross-entropy as the loss function. Fine-tuning was carried out for a maximum of 100 epochs with early stopping criteria implemented to prevent overfitting. For all pre-trained architectures, the final classification layer was modified to output two labels (i.e., cooling or control), corresponding to the cooling prediction task.

#### Model Explainability.

To understand the decision-making processes of the fine-tuned CNN models, Gradient-weighted Class Activation Mapping (Grad-CAM) visualization was used to understand the important features within the images.^33^ Grad-CAM analysis highlights the image regions that most strongly influence (i.e., largest gradients of the model’s output in the final convolution layer) on the cooling detection. As such, it provides insight into how the CNN processes thermal image features and whether the CNN is focusing on clinically relevant anatomical features or relying on spurious correlations.

### Evaluation Setup

We assessed the performance of optical and thermal imaging modalities for cooling region detection using a cross-validation framework. A stratified, patient-level 5-fold cross-validation approach was implemented with two key objectives: (1) maintaining balanced skin tone group representation across all folds, and (2) preventing data leakage by ensuring individual patients’ images appeared exclusively in either training or testing sets within each fold.

To facilitate comparative analysis between imaging modalities and CNN architectures, we maintained a synchronized partitioning strategy with identical patient assignments. Specifically, corresponding optical and thermal image pairs from the same patient were consistently allocated to matching folds (e.g., if Patient A’s optical images were assigned to Fold 1, their thermal images were similarly placed in Fold 1 of the thermal dataset). This parallel stratification ensured that each fold contained identical participant cohorts across both modalities and architectures, thereby eliminating confounding variables and enabling unbiased performance comparisons. Following standard cross-validation methodology, four folds were utilized for model fine-tuning while the remaining fold served as the held-out test set for performance evaluation. Model performance was assessed using four established classification metrics, accuracy, sensitivity, specificity, and F1 score, to provide a multidimensional evaluation of classification capability.

## Results

### Performance Comparison Across Imaging Modalities

#### Overall Performance Comparison

Table 3 summarizes the performance evaluation between optical and thermal imaging modalities for cooling detection across the three CNN architectures. Thermal imaging consistently achieved better performance, with overall accuracies ranging from 0.900 to 0.996 across the three architectures, outperforming optical imaging by 40–50 percentage points. InceptionNetV3 achieved the highest overall performance on thermal images (accuracy = 0.996 ± 0.006, F1 = 0.996 ± 0.006), followed closely by MobileNetV2 (accuracy = 0.990 ± 0.009), and ResNet50 (accuracy = 0.986 ± 0.009). In contrast, optical imaging performance was lower and more variable across all architectures. Overall accuracies were consistently poor, ranging from 0.520 (ResNet50) to 0.547 (MobileNetV2), with high standard deviations indicating significant variability across cross-validation folds. The improved performance on thermal imaging was consistent across all four evaluation metrics, with thermal models achieving near-perfect sensitivity (0.981–0.995) and specificity (0.989–1.000) compared to optical models’ moderate sensitivity (0.486–0.662) and specificity (0.418–0.608).

##### Statistical Analysis.

Paired t-tests confirmed the significance of these observed performance differences between imaging modalities across all CNN architectures. The magnitude of thermal imaging’s improvement was statistically significant and consistent across models: InceptionNetV3 showed the largest performance gap with a mean difference of −0.434 (95% CI: −0.456, −0.412; p < 0.001; Cohen’s d = −27.67), followed by ResNet50 with a mean difference of −0.436 (95% CI: −0.460, −0.412; p < 0.001; Cohen’s d = −24.80), and MobileNetV2 with a mean difference of −0.422 (95% CI: −0.462, −0.382; p < 0.001; Cohen’s d = −14.71). All effect sizes were exceptionally large, indicating not only statistical significance but also practical significance. The p-values across all architectures ranged from 6.39 × 10^−7^ to 7.94 × 10^−6^, providing strong evidence against the null hypothesis of equal performance between modalities.

#### Performance Across Skin Tone Groups

##### Thermal Imaging Performance by Skin Tone.

Thermal imaging performance demonstrated slight variation across skin tone groups, with MST 6 Group and MST 7–10 Group consistently yielding better performance compared to MST 1–5 Group. InceptionNetV3 achieved perfect performance for MST 6 Group and MST 7–10 Group, and MobileNetV2 got perfect specificity on all three groups. Even for MST 1–5 Group, thermal imaging maintained excellent performance, with accuracies ranging from 0.975 (MobileNetV2) to 0.983 (InceptionNetV3 and ResNet50). Notably, all CNN models fine-tuned on thermal images maintained perfect or near-perfect specificity across skin tone groups, indicating excellent ability to correctly identify non-cooling regions regardless of skin tone.

##### Optical Imaging Performance by Skin Tone.

Optical imaging had less consistent findings across the various skin tone groups. Similar to thermal imaging, for both MobileNetV2 and InceptionNetV3, there was improved performance on MST 6 Group and MST 7–10 Group. However, for ResNet50, this trend was reversed with MST 1–5 Group yielding higher accuracy and F1. Although there was limited variation across the skin tones, the accuracies across all models were consistently poor, ranging from 0.518 (ResNet50) to 0.548 (InceptionNetV3). However, higher standard deviations in sensitivity and specificity metrics suggest unstable performance across different patient populations and image acquisition conditions.

### Protocol-Specific Performance Analysis

We performed the protocol analysis for MobileNetV2 as it combines lightweight computational requirements with reasonable performance characteristics. Its position between InceptionNetV3 (highest performance) and ResNet50 (lowest performance) as suggested in Table 3 makes it a suitable representative model for evaluating how imaging protocols affect CNN-based cooling detection across diverse settings.

#### Optical Imaging

Table 4 summarizes the effect of each imaging protocol on both imaging modalities. For optical image classification, there are notable variations in misclassification patterns across the 12 protocols. Protocol 8 (knees stacked, ring light, and 50 cm) exhibited the highest number of misclassifications with 74 errors (9.7% of total optical misclassifications), while Protocol 12 (knees back, ring light, and 50 cm) had the fewest with 59 errors (7.8%). The remaining protocols showed relatively consistent misclassification patterns, with most protocols contributing between 8.0–8.7% of total optical errors.

Despite this protocol-dependent variation in error distribution, optical imaging performance remained consistently suboptimal across all configurations. The total of 761 misclassifications across all protocols demonstrates the fundamental limitations of optical imaging for cooling detection, regardless of specific imaging conditions. No single protocol emerged as clearly superior, with misclassification counts ranging from 59 to 74 images across the 12 different configurations.

#### Thermal Imaging

In contrast to optical imaging, thermal imaging demonstrated remarkable consistency across all imaging protocols as shown in Table 4. Accuracy rates consistently exceeded 90% across all configurations, with the majority of protocol-skin tone combinations achieving perfect (100%) classification. MST 6 Group showed the lowest variability, achieving perfect performance across 11 of 12 protocols with only 2 misclassifications. Even the least consistent group, MST 1–5 Group, maintained accuracies above 90%, with 8 of 12 protocols achieving perfect classification. In terms of protocols, protocol 3 (knees forward, ring light, and 35 cm) had the highest number of misclassified images with 2 for MST 1–5 Group and 2 for MST 6 Group.

#### Camera Type Performance Based on Protocol

Given that imaging performance can vary significantly between camera models, we examined misclassification patterns across the two thermal camera types, FLIR E8XT and ONE^®^ Pro, used in our study for both image modalities (see Table 5). This addresses whether the observed protocol effects are consistent across different imaging devices or represent camera-dependent artifacts.

##### Camera-Specific Modality Performance Analysis.

Analysis of imaging performance across FLIR E8XT and ONE^®^ Pro cameras revealed distinct modality-dependent patterns. For optical imaging, both camera models demonstrated consistently poor performance with substantial protocol sensitivity, though with different optimal configurations. The FLIR E8XT showed better performance with knee back positioning and ring lighting, while the ONE^®^ Pro exhibited less distance sensitivity but greater variability across posture configurations. Notably, both cameras produced comparable overall misclassification rates, indicating that poor optical performance may not be due to specific hardware limitations but rather fundamental modality constraints.

##### Skin Tone Group Variations by Camera Type.

The distribution of misclassifications across skin tone groups differed between camera systems in optical imaging. The FLIR E8XT misclassified 69 images from MST 6 Group compared to ONE^®^ Pro’s 86, while both cameras performed similarly for light MST 1–5 Group and MST 7–10 Group. These variations suggest that camera-specific characteristics may interact differently with skin tone reflectance properties, though the overall poor performance indicates that no camera configuration adequately addresses optical imaging limitations for cooling detection.

##### Thermal Imaging Consistency.

In contrast to optical imaging, thermal imaging demonstrated remarkable consistency across all imaging protocols as shown in Table 4. With only 16 total misclassifications across all protocols, thermal imaging achieved exceptional performance regardless of imaging configuration. Most protocols achieved perfect or near-perfect classification, with 6 of the 12 protocols (Protocols 6, 7, 9, and 10) producing zero misclassifications.

Although there is minimal variation between protocols, Protocol 3 (knees forward, ring light, and 35 cm) had the highest number of misclassified images with 4 errors (25.0% of total thermal misclassifications). Protocols 1, 2, 4, 5, and 11 each contributed 2 misclassifications (12.5% each), while Protocols 8 and 12 had only 1 misclassification each (6.3%). The total of 16 misclassifications across all 1,680 thermal images represents an overall accuracy exceeding 99%, demonstrating thermal imaging’s robustness to protocol variations.

### Error Analysis on Misclassified Thermal Images

**Figure 2.**
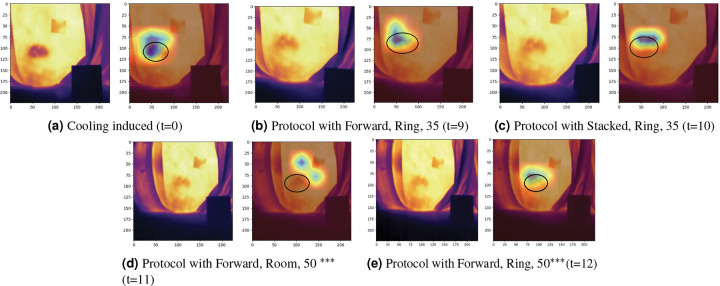
The progression of thermal images for the cooling protocol of a MST 7–10 Group subject and their associated Grad-CAM visualization. For each set of thermal images, the left displays the original image and the right overlays the region with the largest impact on the prediction. The number in parenthesis (t=x) indicates the image acquisition sequence number. *** represents the misclassified images and black circles represents the region of interest.

**Figure 3.**
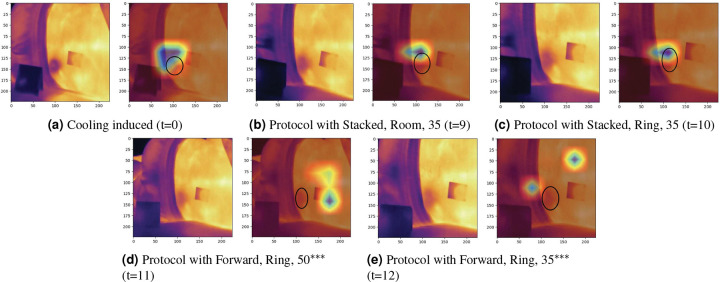
The progression of thermal images for the cooling protocol of a MST 1–5 Group subject and their associated Grad-CAM visualization. For each set of thermal images, the left displays the original image and the right overlays the region with the largest impact on the prediction. The number in parenthesis (t=x) indicates the image acquisition sequence number. *** represents the misclassified images and black circles represents the region of interest.

To understand the sources of misclassification in thermal imaging, we conducted detailed error analyses using Grad-CAM visualizations on two representative cases – one from MST 1–5 Group and one from MST 7–10 Group. Grad-CAM heatmaps overlay blue regions on the thermal image to indicate areas where MobileNetV2 focused its attention during classification, with red hues indicating stronger model attention and blue regions representing less important areas. The thermal images utilize standard color mapping where warmer temperatures correspond to brighter colors (white, yellow, orange) and cooler temperatures to darker colors (purple, blue, black), with baseline skin temperatures differing between the selected subjects (Figure 2 showing higher overall temperatures than Figure 3). Both cases were captured using the FLIR E8XT camera, which exhibits greater temperature sensitivity and thermal contrast compared to the ONE^®^ Pro.^34^ This enhanced thermal range makes the E8XT particularly suitable for identifying subtle temperature variations that could influence model classification behavior.

Although InceptionNetV3 achieved the highest overall accuracy (Table 3), we focus on MobileNetV2 misclassifications as they encompass all failure cases observed across both architectures, providing comprehensive insight into the failure points that can affect CNN-based thermal cooling detection. The following analysis examines how temporal changes in thermal distributions and skin tones impact CNN attention patterns and classification outcomes.

#### MST 7–10 Group Subject Analysis.

Figure 2 presents the image collection sequence for a participant in MST 7–10 Group, illustrating the evolution of thermal patterns after the cooling protocol. The first cooling-induced image (Figure 2a) illustrates the uniform cooler temperatures across the participant’s right posterior superior iliac spine region. The corresponding Grad-CAM visualization (right image of Figure 2a) confirms that MobileNetV2 correctly focused on this cooling region, as evidenced by the concentrated right attention patterns (darker overlay regions) aligned with the thermal cooling zone. As the image acquisition progressed from t=9 to t=12 (Figures 2b–2e), localized skin warming developed within the cooling region, creating isolated thermal hot spots that became increasingly pronounced at t=11 and t=12. Grad-CAM overlay patterns demonstrate that MobileNetV2 focuses on the genuine cooling region for the correctly classified images captured at earlier time points (t=9 and t=10, Figures 2b and 2c, respectively). However, MobileNetV2’s focus shifted to these warming artifacts nearby the cooling region at the next image time point, Figure 2d which lead to misclassification. Surprisingly, for Figure 2e, the Grad-CAM heatmap overlay returns back to a similar region to Figure 2c albeit at less intensity (the blue is lighter). The temperature gradient within the region where Grad-CAM focuses appears to be reduced in this case, diminishing the thermal contrast available to guide model classification, though sufficient gradient remains to direct some attention to the correct region.

#### MST 1–5 Group Subject Analysis.

The image collection sequence after the cooling protocol for a participant from MST 1–5 Group is shown in Figure 3. Similar to the MST 7–10 Group participant, the cooling-induced baseline (Figure 3a) exhibits the uniform cooling region on the participant’s right posterior superior iliac spine region. The last two image acquisitions (Figures 3d and 3e)) were also incorrectly classified. However, unlike the MST 7–10 Group subject, areas closer to the spine and the left paraspinal region exhibited increased skin temperatures. As a result, MobileNetV2 shifted to these warm regions rather than the cooling region with diminishing thermal contrast, as suggested by the Grad-CAM analysis. Interestingly, the preceding correctly classified image at t=10 (Figure 3c) only differs in collection protocol from Figure 3e in terms of knee position (stacked versus forward). This minimal protocol difference (barely perceptible to the human eye other than a slight skew in body position) suggests that the paraspinal warming pattern, rather than the postural variation, is the primary misclassification factor.

#### Cross-Subject Pattern Identification.

Comparative analysis of both subjects revealed consistent error patterns across two different skin tone groups. In both cases, MobileNetV2 correctly classified images when thermal gradients were consistently high in both subjects early in the image acquisition sequence, but the few misclassifications occurred as the thermal gradient reduced over time. Grad-CAM visualizations demonstrated that the model’s attention is impacted by warmer and higher contrast regions rather than spatially distributed cooling patterns during misclassification events. Despite different anatomical locations of warming (paraspinal regions in the MST 1–5 Group subject versus warm spots on or near the cooling region in the MST 7–10 Group subject), the attention-shifting behavior was comparable across both cases, demonstrating subject-independent model behavior rather than individual subject variation.

## Discussion

This study systematically evaluated the impact of imaging protocol variations on the reliability of deep learning models trained to detect temperature changes. Our results establish the robustness of thermal imaging-based deep learning models across diverse imaging conditions, achieving >98% accuracy across 12 different protocols compared to 52–55% for optical imaging. While thermal imaging’s superior performance for temperature detection might be expected given its direct measurement of thermal radiation, this systematic comparison was necessary to quantify the performance gap and establish protocol robustness across different CNN architectures. Importantly, thermal imaging demonstrated consistent protocol independence across all three CNN architectures, while optical imaging performance varied substantially across protocols. This pattern held regardless of architecture complexity, indicating that the protocol robustness of thermal imaging is a characteristic of the imaging modality rather than an artifact of specific CNN architectures. This finding has implications for clinical deployment, as thermal imaging-based models can be expected to maintain performance across varied imaging conditions regardless of the chosen CNN architecture.

The robustness of thermal imaging model performance also extends across diverse skin tones. InceptionNetV3 achieved 100% accuracy for MST 6 Group and MST 7–10 Group, while maintaining 98.3% accuracy for MST 1–5 Group. This consistency indicates that thermal imaging performed reliably across the skin tone variations tested, contrasting with documented challenges faced by optical-based systems. All three CNN architectures demonstrated similar skin tone independent-performance patterns, with InceptionNetV3 showing marginally better results. In contrast, optical imaging achieved moderate accuracy (32–55%) across all skin tone groups and architectures, with overall accuracies of 54.7±0.020% (MobileNetV2), 54.0±0.002% (InceptionNetV3), and 52.0±0.020% (ResNet50). The large effect sizes (Cohen’s d: 14.71–27.67) also indicate meaningful practical differences between modalities. While CNNs trained on thermal images should be better at identifying controlled cooling than optical images, these findings provide a critical foundation for thermal imaging’s potential in clinical pressure injury detection, given that temperature changes often precede visible skin changes, particularly in patients with darker skin tones where erythema may be difficult to detect visually.^7^

Analysis of misclassification patterns revealed distinct failure modes between imaging modalities. Thermal imaging misclassifications were primarily attributed to temporal rewarming effects, with the presence of localized warm regions and diminished temperature gradients impacting model performance. Grad-CAM visualizations showed that attention either shifted toward these warm spots in later image acquisitions or focused on regions with reduced temperature gradients, leading to misclassification as control cases. These failures occurred consistently across skin tone groups and were independent of imaging protocol variations. In contrast, optical imaging misclassifications showed protocol-dependent patterns, with performance varying based on lighting conditions, patient positioning, and camera distance. While literature documents optical imaging bias against darker skin tones in medical applications,^35^ our results show universal inadequacy for temperature detection across all skin tones, highlighting the potential value for thermal imaging.

Our error analysis revealed important implications for thermal imaging dataset development as clinical validation moves forward. The emergence of thermal artifacts during natural rewarming processes suggests the importance of careful label curation when training and deploying thermal imaging models for pressure injury detection. In the context of pressure injury detection, where images may contain both warm and cool regions,^36^ single image-level labels may be insufficient, suggesting that region segmentation and annotation approaches may be necessary to accurately capture the spatial distribution of thermal patterns and improve model performance for clinical applications. The CNN model’s ability to detect anomalies in both cool and warm regions, despite being trained on only cool images, shows promise since pressure injury detection, which requires awareness of both temperature variations.^36^ Furthermore, the CNN model confusion with thermal images containing mixed thermal signatures may actually indicate the need to include such cases in training datasets. The pronounced temperature differences in our controlled cooling protocol may not reflect the subtle thermal variations typically encountered clinically, suggesting that training on images with less distinct thermal patterns could improve real-world performance.

Several limitations should be considered when interpreting our findings. This study used controlled cooling simulations rather than actual pressure injury development, which may limit the generalizability of thermal imaging’s apparent superiority to clinical scenarios. The healthy adult cohort may not reflect clinical populations with comorbidities and physiological variations that could influence thermal imaging performance. Additionally, the collection protocol resulted in limited sample sizes within MST 1–5 Group and MST 6 Group, which may reduce precision for protocol-specific recommendations. Future validation in clinical populations with actual pressure injury risk and larger balanced cohorts across all skin tone groups are essential to confirm the clinical utility of thermal imaging approaches. Future research should also systematically assess whether medical shortcuts occur in thermal imaging interpretation, examining how providers integrate AI-generated assessments with clinical judgment. Understanding whether thermal imaging truly reduces cognitive biases or creates new technology-related shortcuts will be crucial for ensuring equitable healthcare delivery.

## Conclusion

This study demonstrates that thermal images serve as a better modality than optical for cooling detection, achieving >98% accuracy with minimal protocol sensitivity compared to optical imaging’s 52–55% accuracy with substantial protocol dependence. The large effect sizes and consistent performance across protocols indicate that thermal imaging has the capability to provide more reliable diagnostic information for early pressure injury detection. Our error analysis revealed important considerations for thermal imaging dataset development, showing that temporal rewarming and reduced thermal gradients can affect model performance and labeling validity. These findings emphasize the importance of inclusive validation in medical device development to ensure equitable performance across diverse patient populations.

The demonstrated robustness of thermal imaging-based deep learning models across diverse skin tones and imaging conditions supports its potential for widespread clinical implementation, particularly given the substantial economic burden of hospital-acquired pressure injuries. These findings establish the foundation needed for clinical validation in actual patient populations. The models’ reliable performance across different imaging protocols and skin tones suggests potential for broader application in scenarios requiring early detection of pressure injuries and other tissue damage.

## Figures and Tables

**Table 1.** Distribution of participants by MST scale

Skin Tone Group	MST Level	Number of participants (%)	Number of Images

MST 1–5 Group	1	0 (0.0)	0
	2	3 (8.6)	144
	3	0 (0.0)	0
	4	1 (2.9)	48
	5	1 (2.9)	48

MST 6 Group	6	7 (20.0)	336

MST 7–10 Group	7	22 (62.9)	1056
	8	1 (2.9)	48
	9	0 (0.0)	0
	10	0 (0.0)	0

**Table 2.** Imaging protocol configurations.

Protocol	Posture			Lighting		Distance (cm)	
		
	Forward	Stacked	Back	Room	Ring	35	50

1	✓			✓		✓	
2	✓			✓			✓
3	✓				✓	✓	
4	✓				✓		✓
5		✓		✓		✓	
6		✓		✓			✓
7		✓			✓	✓	
8		✓			✓		✓
9			✓	✓		✓	
10			✓	✓			✓
11			✓		✓	✓	
12			✓		✓		✓

**Table 3.** CNN model performance for cooling detection across skin tone groups and imaging modalities.

Modality	Model	Skin Tone Group	Accuracy	F1	Sensitivity	Specificity

Optical	MobileNetV2	MST 1–5 Group	0.525	0.374	0.283	0.767
		MST 6 Group	0.539	0.563	0.595	0.482
		MST 7–10 Group	0.539	0.563	0.595	0.482

		Overall	0.547±0.020	0.500±0.085	0.486±0.191	0.608±0.220

	InceptionNetV3	MST 1–5 Group	0.542	0.580	0.633	0.450
		MST 6 Group	0.548	0.556	0.565	0.530
		MST 7–10 Group	0.548	0.556	0.565	0.530

		Overall	0.540±0.002	0.575±0.084	0.662±0.205	0.418±0.203

	ResNet50	MST 1–5 Group	0.542	0.601	0.692	0.392
		MST 6 Group	0.518	0.547	0.583	0.452
		MST 7–10 Group	0.518	0.547	0.583	0.452

		Overall	0.520±0.020	0.505±0.144	0.576±0.299	0.464±0.325

Thermal	MobileNetV2	MST 1–5 Group	0.975	0.974	0.950	1.000
		MST 6 Group	0.994	0.994	0.988	1.000
		MST 7–10 Group	0.994	0.994	0.988	1.000

		Overall	0.990±0.009	0.990±0.010	0.981±0.019	1.000±0.000

	InceptionNetV3	MST 1–5 Group	0.983	0.983	0.975	0.992
		MST 6 Group	1.000	1.000	1.000	1.000
		MST 7–10 Group	1.000	1.000	1.000	1.000

		Overall	0.996±0.006	0.996±0.006	0.995±0.007	0.996±0.005

	ResNet50	MST 1–5 Group	0.983	0.983	0.967	1.000
		MST 6 Group	0.991	0.991	0.994	0.988
		MST 7–10 Group	0.991	0.991	0.994	0.988

		Overall	0.986±0.009	0.986±0.009	0.983±0.015	0.989±0.021

Overall values represent mean ± standard deviation from the 5-fold cross-validation.

**Table 4.** Analysis of the number of misclassified images of MobileNetV2 based on protocol.

	Protocol												
		
Modality	1	2	3	4	5	6	7	8	9	10	11	12	Total

**Optical (%)**	63 (8.3)	64 (8.4)	63 (8.3)	63 (8.3)	61 (8.0)	62 (8.1)	66 (8.7)	74 (9.7)	63 (8.3)	60 (7.9)	63 (8.3)	59 (7.8)	**761 (100.0)**

**Thermal (%)**	2 (12.5)	2 (12.5)	4 (25.0)	2 (12.5)	2 (12.5)	0 (0.0)	0 (0.0)	1 (6.3)	0 (0.0)	0 (0.0)	2 (12.5)	1 (6.3)	**16 (100.0)**

**Table 5.** Misclassified images by camera type, image modality, and protocol using MobileNetV2.

			Optical			Thermal		
		
Camera type	Protocol		1–5	6	7–10	1–5	6	7–10

**FLIR E8XT**	**Posture**	Knee forward	22	28	82	3	1	3
		Knee stacked	18	23	84	0	0	1
		Knee back	16	18	83	1	0	0

	**Lighting**	Room	30	36	120	1	0	3
		Ring	26	33	129	3	1	1

	**Distance**	35cm	32	31	121	2	1	2
		50cm	24	38	128	2	0	2

**ONE^®^ Pro**	**Posture**	Knee forward	17	30	74	1	1	1
		Knee stacked	20	29	89	0	0	2
		Knee back	21	27	80	1	0	1

	**Lighting**	Room	29	44	114	0	0	2
		Ring	29	42	129	2	1	2

	**Distance**	35cm	28	44	123	2	1	2
		50cm	30	42	120	0	0	2

## Data Availability

Due to participant privacy concerns, the optical and thermal image dataset generated during this study is limited to those who request access, have sufficient CITI certification, sign confidentiality and data-use agreements, and have IRB approval at their institution. Data requests should be directed to Sharon Eve Sonenblum.
